# Contents of US Food and Drug Administration Refuse-to-File Letters for New Drug Applications and Efficacy Supplements and Their Public Disclosure by Applicants

**DOI:** 10.1001/jamainternmed.2020.8866

**Published:** 2021-02-15

**Authors:** Harinder Singh Chahal, Sanjana Mukherjee, Daniel W. Sigelman, Robert Temple

**Affiliations:** 1 Public Health Strategy and Analysis Staff, Office of the Commissioner Food and Drug Administration, Silver Spring, Maryland; 2 Office of New Drugs, Center for Drug Evaluation and Research Food and Drug Administration, Silver Spring, Maryland

## Abstract

**Importance:**

Before reviewing drug applications, the US Food and Drug Administration (FDA) conducts “filing reviews” to assess whether they are complete enough for full review. If the applications are incomplete, the FDA issues refuse-to-file (RTF) letters identifying deficiencies. The FDA does not make these RTF letters public at the time of issuance. Why the FDA issues RTF letters and how often the letters and their contents are made publicly available are unknown.

**Objectives:**

To quantitatively analyze the FDA’s reasons for issuing RTF letters and assess the public transparency of RTF letters and their contents.

**Design and Setting:**

This cross-sectional study analyzes RTF letters issued in response to new drug applications and efficacy supplements (applications for new indications or patient populations for already approved drugs) submitted to the FDA between January 1, 2008, and December 31, 2017. Statistical analysis was conducted in July 2019.

**Main Outcomes and Measures:**

Two types of information were extracted and cataloged from RTF letters: (1) the reasons why the FDA refused to file applications and (2) the FDA comments that, while not a basis for RTF letters, conveyed important information to applicants. The extent to which applicants publicly disclosed the FDA’s refusal reasons were also assessed.

**Results:**

The study included 103 RTF letters containing a total of 644 identified FDA refusal reasons. Among the 2475 applications that the FDA received during the study time frame, 98 (4.0%) received RTF letters. Overall, 84.5% (544 of 644) of the refusal reasons were for scientific deficiencies; most reasons were related to drug efficacy and safety (196 [30.4%]) and drug quality (125 [19.4%]). The remaining 15.5% of refusal reasons (100 of 644) were for application organization deficiencies or legal issues. A total of 26.2% of the RTF letters (27 of 103) identified presubmission advice from the FDA that applicants did not follow; the most frequently ignored advice was related to clinical trial design (33.3% [9 of 27]), followed by product chemistry and manufacturing (25.9% [7 of 27]). Applicants publicly disclosed the existence of 16 of 103 RTF letters (15.5%); however, only 5.4% of applicant-disclosed reasons (35 of 644) matched the refusal reasons that the FDA had provided in the RTF letters.

**Conclusions and Relevance:**

This cross-sectional study found that the FDA refused to file applications for substantive reasons related to quality, safety, and efficacy, and applicants’ disclosure of those reasons was incomplete. This work sheds light on the FDA’s regulatory decision-making processes and the RTF reasons that could delay availability of therapies to patients.

## Key Points

**Question:**

What are the US Food and Drug Administration’s (FDA’s) reasons for issuing refuse-to-file (RTF) letters to drug applicants, and to what degree do these applicants publicly disclose the issuance and contents of RTF letters?

**Findings:**

This cross-sectional study found that 84.5% of the FDA’s refusal reasons (544 of 644) were due to scientific deficiencies; most reasons related to drug efficacy and safety (196 [30%]) and drug quality (125 [19%]). Applicants publicly disclosed the issuance of only 16% of RTF letters (16 of 103) and disclosed only 5% of the refusal reasons (35 of 644) that the FDA cited in the letters.

**Meaning:**

The FDA refuses to file applications for substantive reasons related to the quality, safety, and efficacy of drug products, and applicants’ public disclosures regarding RTF letters are incomplete.

## Teaser

This cross-sectional study analyzes the US Food and Drug Administration’s reasons for issuing refuse-to-file letters and assesses public transparency of refuse-to-file letters and their contents.

## Introduction

To market drugs in the United States, companies must submit applications to the US Food and Drug Administration (FDA) containing evidence to support their drugs’ safety, efficacy, and quality. Within 60 days of application submissions, the FDA conducts filing reviews to assess whether applications are complete enough to be fully reviewed. Applicants found to have submitted incomplete applications are issued refuse-to-file (RTF) letters identifying the reasons preventing full reviews. Filing reviews allow the FDA to avoid the expenditure of resources on incomplete applications and allow applicants to address application issues early to potentially avoid multiple review cycles or nonapproval.^1^ After receiving RTF letters, applicants have the right to “file over protest” and ask the FDA to conduct full reviews of their original submissions.^2^

Under current statutes and regulations, the FDA is generally prohibited from disclosing the existence or contents of RTF letters for unapproved applications or supplemental applications. On application approval, the FDA sometimes posts an approval package to its website, generally including any RTF letters, although there may be redactions. Applicants may disclose this information at any time; however, how often they do so is not known. Previous research on FDA-issued complete response letters showed that applicants’ disclosure of the reasons underlying the FDA’s decisions not to approve new drugs are often limited.^3^

Previous RTF analyses have relied on limited, publicly available information to understand the FDA’s refusal reasons.^4,5,6,7,8^ We studied the FDA’s reasons for issuing RTF letters and systematically assessed the degree of public transparency of RTF letters and their contents.

## Methods

### Study Design

We designed a cross-sectional study to analyze RTF letters issued to selected drug applications submitted to the FDA’s Center for Drug Evaluation and Research between January 1, 2008, and December 31, 2017. Our analysis deidentified and aggregated the data to prevent identification of applicants or specific drugs. As it did not involve clinical data or human participants, this study was not submitted for ethics review.

### Collection of RTF Letters and Applicant Information

The FDA’s internal database was used to collect RTF letters and application-level information (eMethods in the Supplement). We included (1) new drug applications (NDAs) for new molecular entities (drugs with active moieties that have not been previously approved or marketed in the US), (2) NDAs for drugs that are not new molecular entities, (3) and efficacy supplements (applications for new indications or patient populations for already approved drugs). Because the FDA could refuse to file applications more than once, the number of RTF letters is greater than the number of refused applications.

### Coding of Content of RTF Letters

To assess the content of RTF letters, we extracted 2 types of information: (1) the reasons the FDA gave for refusing to file applications and (2) additional comments that, while conveying important information or suggestions to applicants, were not a basis for FDA’s refusal to file (ie, non-RTF reasons).

Reasons were categorized into the specific deficiencies the FDA identified in these applications. In an RTF letter, a reason could be parsed from a phrase, sentence, or longer discussion of a single deficiency. Each reason was assigned to 1 of 7 domains—general scientific and technical; application organization and legal; clinical efficacy; clinical safety; chemistry, manufacturing, and controls; clinical pharmacology and biopharmaceutics; and nonclinical—as well as to 1 subdomain within each domain. All domains other than application organization and legal consist of scientific or technical reasons given to applicants for refusing to file applications (see eTable 1 in the Supplement for all domains and subdomains).

Comments (non-RTF reasons) were coded into 5 categories: an observation that the sponsor had not heeded presubmission requests or advice from the FDA (these were further subdivided into 15 types of specific advice), discussion of specific application shortcomings that might cause issues during full review, recommendations for specialized consultation to improve applications, recommendation that applicants meet with FDA reviewers, and comparisons with other drugs in similar therapeutic areas (eTable 2 and eTable 3 in the Supplement).

### Public Disclosure of RTF Letters and RTF Content

We examined applicants’ disclosures after the FDA issued the RTF letters but before drug approval. To assess applicants’ RTF disclosures, we conducted searches in press releases or US Securities and Exchange Commission regulatory filings (for US publicly traded companies), starting from the RTF issue dates to July 2019, using publicly available sources (eMethods in the Supplement).^9,10,11,12,13^ We compared applicants’ public descriptions of why they received RTF letters with the FDA’s stated reasons. A general or partial mention of the FDA’s refusal reasons or non-RTF comments was deemed sufficient to constitute public disclosure. We also assessed how often the FDA, pursuant to applicable requirements and policies, disclosed the RTF letters after application approval (eTable 4 in the Supplement); to do this, we used Drugs@FDA, the FDA’s public database that contains drug approval packages.^14^

### Statistical Analysis

Two investigators independently coded all RTF letters, press releases, and Securities and Exchange Commission filings. The investigators had a 91% convergence in coding; discrepancies were resolved by consensus. To capture as many resubmissions and disclosures as possible, we assessed the regulatory status of applications in July 2019, 1.5 years after the study period closed. We analyzed 2 primary units: the RTF letters as a whole and the reasons for refusal to file that the FDA articulated in the RTF letters (eMethods in the Supplement).

## Results

During the study period, the FDA received 2475 applications eligible for inclusion in this study; 1180 were NDAs and 1295 were efficacy supplements. Overall, 4.0% of applications (98) received RTF letters—6.2% of NDAs (73 of 1180) and 1.9% of efficacy supplements (25 of 1295). The study included 103 RTF letters issued in response to these 98 applications; 76 RTF letters were issued to 73 NDAs (3 NDAs received 2 RTF letters each) and 27 RTF letters were issued to 25 efficacy supplements (2 supplements received 2 RTF letters each) (Table).

**Table.** Characteristics of RTF Letters, FDA-Issued Reasons for Refusals, and Public Disclosure by the Applicants

Characteristic	No./total No. (%)		RR (95% CI)	RTF letters with ≥1 disclosed reason (n = 11), No./total No. (%)^b^	RR (95% CI)	No./total No. (%)		RR (95% CI)
	All FDA-issued RTF letters (N = 103)^a^	Publicly disclosed RTF letters by applicants (n = 16)^b^				All FDA reasons in RTF letters (N = 644)^a^	Publicly disclosed reasons by applicants (n = 35)^b^	
Application type								
Original NDA	76/103 (73.8)	14/76 (18.4)	2.49 (0.60-10.23)	11/76 (14.5)	NA	462/644 (71.7)	35/462 (7.6)	NA
Efficacy supplement	27/103 (26.2)	2/27 (7.4)		0		182/644 (28.3)	0	
Original NDA submission type								
NME	20/76 (26.3)	7/20 (35.0)	2.80 (1.13-6.99)	5/20 (25.0)	2.33 (0.79-6.81)	202/462 (43.7)	22/202 (10.9)	2.18 (1.12-4.22)
Non-NME	56/76 (73.7)	7/56 (12.5)		6/56 (10.7)		260/462 (56.3)	13/260 (5.0)	
Applicant company size								
Large (>1250 employees)	51/103 (49.5)	5/51 (9.8)	0.45 (0.17-1.21)	1/51 (2.0)	0.10 (0.01-0.75)	235/644 (36.5)	2/235 (0.9)	0.10 (0.02-0.43)
Small (<1250 employees)	51 (49.5)	11/51 (21.6)		10/51 (19.6)		408/644 (63.4)	33/408 (8.1)	
Unknown^c^	1/103 (1.0)	0	NA	0	NA	1/644 (0.2)	0	NA
Applicant public or private status								
Publicly traded in the US	42/103 (40.8)	14/42 (33.3)	10.17 (2.44-42.42)	9/42 (21.4)	6.53 (1.49-28.74)	194/644 (30.1)	20/194 (10.3)	3.09 (1.61-5.91)
Private	35/103 (34.0)	1/35 (2.9)^d^	0.13 (0.02-0.94)	1/35 (2.9)	0.19 (0.02-1.46)	273/644 (42.4)	5/273 (1.8)	0.23 (0.09-0.58)
Foreign (not US traded)^e^	26/103 (25.2)	1/26 (3.8)	0.19 (0.03-1.42)	1/26 (3.8)	0.29 (0.42-2.20)	177/644 (27.5)	10/177 (5.6)	1.05 (0.52-2.15)

Abbreviations: FDA, US Food and Drug Administration; NA, not applicable; NDA, new drug application; NME, new molecular entity; RR, relative risk; RTF, refuse-to-file.

^a^ Column percentage.

^b^ Row percentage.

^c^ The 1 unknown value was not used in RR calculations.

^d^ The private company announced the existence of the RTF letter in a US Securities and Exchange Commission filing in anticipation of trading stock on a public exchange.

^e^ Regulatory filings with foreign regulators not assessed for foreign firms that were not traded in the US.

Of the 98 applications, 54.1% (53 [47 NDAs and 6 efficacy supplements]) had been resubmitted after the RTF letters were received and the FDA’s refusal reasons were potentially addressed. The median time to resubmission after RTF letters was 182 days (interquartile range [IQR], 100-460 days), with 16 applications taking more than 1 year to resubmit. Of the 53 that were resubmitted, 71.7% (38 [34 NDAs and 4 efficacy supplements]) were approved at time of analysis within a median of 784 days (IQR, 529-1283 days) from the original submission date; approval of 21 applications took more than 2 years. This approval time includes all intermediate regulatory steps, including the time applicants took to address the RTF letter and potential multiple review cycles after FDA nonapproval. Four of 53 applications had been withdrawn and 3 received another RTF letter. Applicants withdrew 6 applications after receiving RTF letters without resubmitting.

Overall, 37 of 98 applications (37.8%) remain in RTF status without resubmissions a median of 2245 days (IQR, 1902-3106 days) after issuance of an initial RTF letter. Four NDAs were filed over protest after receipt of RTF letters within a median of 165.5 days (IQR, 83-302 days); after full review, none were approved.

### FDA’s Reasons for Issuing RTF Letters

**Figure 1.**
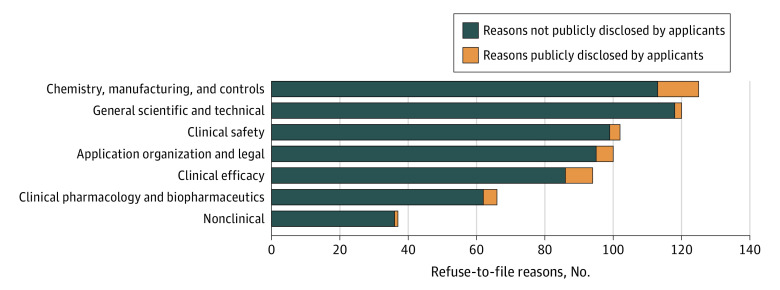
Number of US Food and Drug Administration–Stated Reasons in Refuse-to-File Letters by Domain and Matching Public Disclosures by the Applicants

Of the 103 FDA-issued RTF letters, 1.9% (2) cited deficiencies in all 7 domains, while 34.0% (35) cited deficiencies in only 1 domain (median, 3 [IQR, 2-4; range, 1-6]). Clinical efficacy deficiencies were seen in 44.7% of RTF letters (46 of 103) and safety deficiencies were seen in 36.9% of RTF letters (38 of 103); deficiencies in both efficacy and safety domains were cited in 26.2% of RTF letters (27 of 103) (Figure 1).

**Figure 2.**
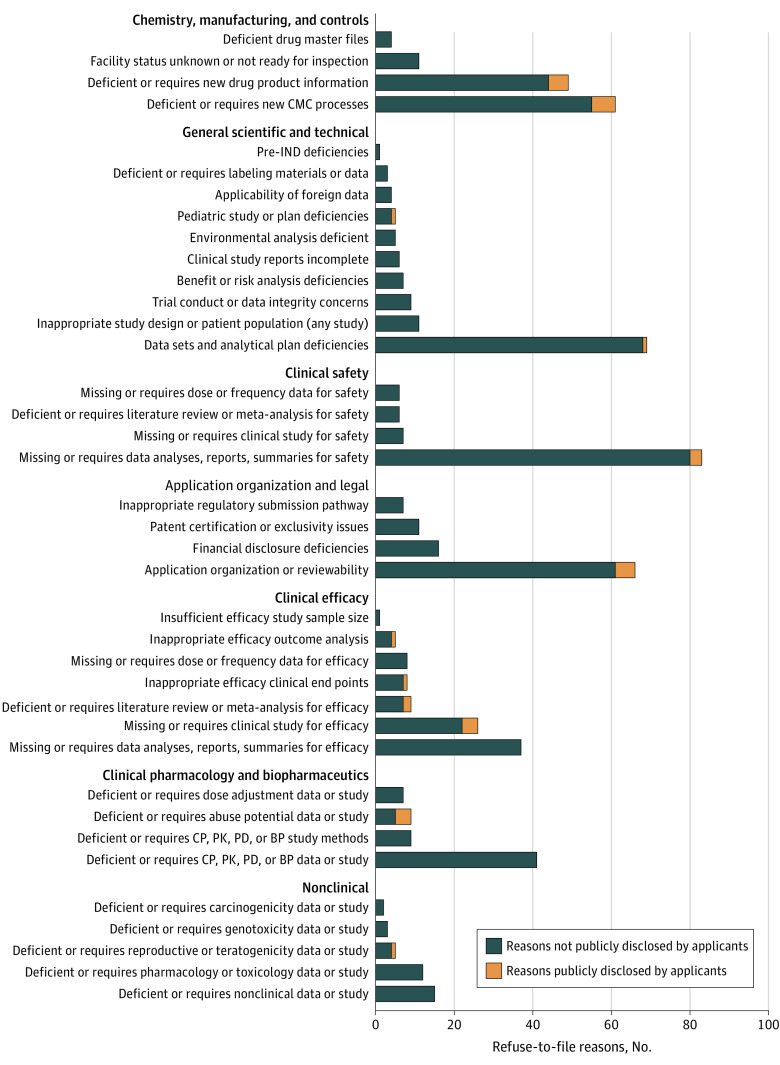
Number of US Food and Drug Administration–Issued Refuse-to-File Reasons by Domain and Subdomain and Matching Public Disclosure by the Applicants BP indicates biopharmaceutics; CMC, chemistry, manufacturing, and controls; CP, clinical pharmacology; IND, investigational new drug application; PD, pharmacodynamics; and PK, pharmacokinetics.

We identified a total of 644 reasons for which the FDA issued RTF letters, with a median of 4 reasons (IQR, 2-6) per letter. Overall, 84.5% of the refusal reasons (544 of 644) referenced scientific deficiencies; the remaining 15.5% of reasons (100 of 644) fell into the application organization and legal domain (Figure 1). The largest portion of refusal reasons related to the chemistry, manufacturing, and controls domain (19.4% [125 of 644]), while clinical safety (15.8% [102 of 644]) and clinical efficacy (14.6% [94 of 644]) reasons collectively accounted for 30.4% (196 of 644) of RTF reasons (see Figure 2 for detailed subdomain data).

### FDA’s Non-RTF Comments

**Figure 3.**
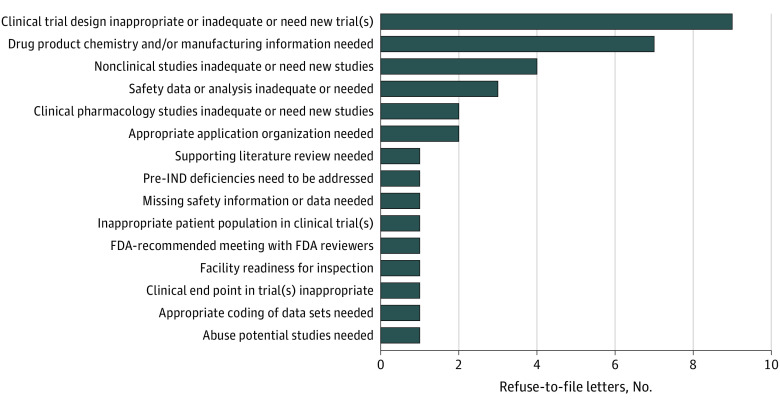
Types of US Food and Drug Administration’s (FDA's) Presubmission Requests or Advice Not Followed by the Applicants IND indicates investigational new drug application.

In 67.0% of RTF letters (69 of 103), the FDA included 89 non-RTF comments; these included 54 comments on specific application shortcomings that might cause issues during full review, 4 recommendations that applicants meet with FDA reviewers, 2 comparisons with other drugs in similar therapeutic areas, and 1 recommendation for specialized consultation to improve applications. In 26.2% of RTF letters (27 of 103), the FDA identified advice or issues raised in presubmission communications that applicants had not followed or addressed in initial application submissions that resulted in RTF letters. The most frequently ignored advice related to clinical trial design (33.3% [9 of 27]), followed by drug chemistry and manufacturing (25.9% [7 of 27]) (Figure 3).

### Public Disclosure of RTF Letters and RTF Content

Overall, applicants disclosed the existence of 15.5% of RTF letters (16 of 103) in press releases or US Securities and Exchange Commission filings within a median of 5 days (IQR, 4-9 days) of receiving RTF letters, but no applicant disclosed a full RTF letter (Table). Of the 38 applications that had been approved at the time of analysis, the FDA had posted redacted RTF letters for 29 (76.3%) in approval packages a median of 34 months (IQR, 21-43 months) after RTF issuance. However, FDA disclosure in approval packages accounts for only 28.2% of study RTF letters (29 of 103). In total, the existence of 35.9% of RTF letters (37 of 103) was eventually made public: 8 by companies, 21 by the FDA, and 8 by both; as of July 2019, 64.1% of the RTF letters (66) remained undisclosed.

Of 16 applicant-disclosed RTF letters, 11 applicants mentioned at least 1 FDA refusal reason in their public acknowledgment of the RTF letters (Table). In these 11 RTF letters, a total of 35 matching reasons, accounting for only 5.4% of the FDA’s 644 reasons (35 of 644) were disclosed (Figure 1). Collectively, applicants disclosed only 5.6% of the FDA’s safety and efficacy reasons (11 of 196). The disclosure rate was lower for FDA comments (non-RTF reasons) at 3.4% (3 of 89); these disclosures related only to FDA comments on application shortcomings that might cause issues during full review.

## Discussion

This study found that FDA refusal to file NDAs and efficacy supplements for review is rare. When applications are refused for review, however, it is for numerous substantive reasons associated primarily with missing information needed to evaluate product quality, safety, and efficacy. Notwithstanding that missing scientific and technical matters accounted for 84.5% of refusal reasons, we found that the public disclosure of these RTF reasons is minimal. Safety and effectiveness assessment lies at the heart of the FDA’s drug review, yet 44.7% of RTF letters were issued owing to missing efficacy data, and 26.2% of RTF letters cited missing both safety and efficacy data. This finding shows that RTF letters address foundational issues in drug review.^15^

An RTF letter has implications for patients as it may cause delays in their access to a drug. In our study, approval of resubmitted applications took about 16 to 18 months longer than the overall approval time for all NDAs and biologic licensing applications the FDA reviewed in 2018.^16^ A direct comparison with applications that did not receive RTF letters was not feasible in this study design, as such analysis requires a cohort study design that matches applications on several characteristics, for example, review priority and number of review of cycles.

One notable finding was that, in 26.2% of RTF letters, the FDA had noted presubmission advice that the applicants ignored in their submissions. The FDA’s presubmission meetings, such as meetings at the end of the phase 2 study or the pre-NDA meeting, as well as other similar communications, afford applicants important opportunities to obtain FDA guidance on the clinical, scientific, and drug quality information needed to support a successful application.^17^ Furthermore, because “FDA reviewers oversee the totality of the preapproval development process, …[they] are in a unique position to help identify common themes and systematic weaknesses across similar products and can draw important lessons from what they see.”^18^^(p13)^ In this study, most commonly ignored advice related to clinical trials and drug chemistry or manufacturing. Given the importance of the need for sound evidence from well-conducted trials and high-quality drugs, ignoring such advice can lead to an RTF letter and delays in application review and subsequent approval of a drug. Thus, it is important for applicants to heed presubmission advice to facilitate timely review of applications and, by extension, potentially speedier patient access to new drugs.

Our study also suggests that exercising the option to file over protest on receipt of an RTF letter may not prove fruitful, as none of the 4 applications filed over protest was approved after full review.^19^ It is thus likely that filing over protest will lead to additional delays in application review, as applicants eventually must rectify the deficiencies. In contrast, 71.7% of applications that addressed the FDA’s concerns in the RTF letters prior to resubmission had been approved at the time of analysis.

Our findings are broadly consistent with previous analyses that found refuse to file reasons associated with deficiencies in clinical information, application formatting and organization, and manufacturing.^6,7^ However, because we analyzed a more comprehensive set of unredacted and nonpublic RTF letters, we cannot directly compare our findings with prior work in this area.

### Transparency of Refusal Reasons and Policy Considerations

Because the FDA does not disclose RTF letters when they are issued, the public must rely on applicants’ disclosures. We found such disclosures to be limited. Applicants’ receipt of 84.5% of RTF letters was not disclosed, while only 5.4% of the FDA’s RTF reasons were disclosed, and then only with minimal detail. Improved disclosure of RTF reasons may help patients, clinicians, and other applicants. For patients and clinicians, knowing that refusals occurred, as well as the reasons (if disclosed with adequate details) underlying them can provide information on the status of development of new therapies.^4^ Other applicants may learn from refusals how to avoid pitfalls in their own applications, but only if disclosures are of sufficient quality, with relevant details.

A 2010 FDA transparency report recommended that the FDA disclose both the existence and content of RTF letters at the time of issuance, which could require amendments to FDA regulations.^4^ To date, the FDA has not implemented this recommendation. The subset of redacted RTF letters that the FDA publishes on applications’ approval in approval packages may be helpful for some stakeholders. However, because these approval packages are typically posted several years after RTF issuance, the RTF letters they contain provide no real-time insights into development cycles of drugs for clinicians and patients. Because applicants are not subject to the confidentiality constraints imposed on the FDA, they could voluntarily make their RTF letters public to help their stakeholders better understand the FDA’s scientific concerns, as well as the regulatory status and prospects of their applications. Although drug applicants disclose to the public their submission of 88.3% submitted NDAs, they rarely disclose other regulatory steps.^20^ In our study, we did not find a single instance in which an applicant publicly released a full RTF letter. Transparency of such regulatory steps can keep clinicians and patients informed on timelines of novel drug approvals.

### Limitations

This study has some limitations. First, the process of identifying and coding refusal reasons can be subjective, limiting reproducibility. However, 2 investigators had a 91% convergence and, given the large number of reasons identified, any subjectivity in coding is unlikely to change the fundamental findings. Second, because we analyzed RTF letters for select NDAs and efficacy supplements, the findings cannot be generalized to other types of FDA applications. Third, our conservative approach to coding, which adhered to the precise terms FDA reviewers used in the letters, may underrepresent safety and efficacy reasons; however, this approach avoids any potential bias in coding reasons. Fourth, as applicants’ minimal or partial mentions of FDA-stated reasons qualified as disclosure, it is possible that we overestimated disclosure.

## Conclusions

We found that the FDA refuses to file applications for substantive reasons related to quality, safety, and efficacy of drugs, and disclosure of the FDA’s refusal reasons is incomplete. Greater transparency of such reasons could help applicants avoid RTF letters and thereby facilitate timelier patient access to new therapies.

## Notes

Supplement. **eMethods.** **eTable 1.** Domains and Sub-Domains Used to Classify the Contents of FDA-Issued RTFs **eTable 2.** Categories of Non-Refuse-to-File Comments FDA Made in RTFs **eTable 3.** Types of FDA Presubmission Requests or Advice Not Followed by Applicants **eTable 4.** Redacted RTFs Included in This Study That FDA Made Publicly Available as of January 2020 **eReferences.**
