# Supplementary material for: Contents of US Food and Drug Administration Refuse-to-File Letters for New Drug Applications and Efficacy Supplements and Their Public Disclosure by Applicants
Source: JAMA Intern Med. 2021 Feb 15;181(4):522–9. doi: 10.1001/jamainternmed.2020.8866 (PMC7885096; doi:10.1001/jamainternmed.2020.8866)
Supplement: Supplement. — eMethods. eTable 1. Domains and Sub-Domains Used to Classify the Contents of FDA-Issued RTFs eTable 2. Categories of Non-Refuse-to-File Comments FDA Made in RTFs eTable 3. Types of FDA Presubmission Requests or Advice Not Followed by Applicants eTable 4. Redacted RTFs Included in This Study That FDA Made Publicly Available as of January 2020 eReferences. [file jamainternmed-e208866-s001.pdf]

Supplementary Online Content

Chahal HS, Mukherjee S, Sigelman DW, Temple R. Contents of US Food and Drug Administration refuse-to-file letters for new drug applications and efficacy supplements and their public disclosure by applicants. *JAMA Intern Med.* Published online February 15, 2021. doi:10.1001/jamainternmed.2020.8866

eMethods.
eTable 1. Domains and Sub-Domains Used to Classify the Contents of FDA-Issued RTFs
eTable 2. Categories of Non-Refuse-to-File Comments FDA Made in RTFs
eTable 3. Types of FDA Presubmission Requests or Advice Not Followed by Applicants
eTable 4. Redacted RTFs Included in This Study That FDA Made Publicly Available as of January 2020
eReferences.

This supplementary material has been provided by the authors to give readers additional information about their work.

eMethods.

Study design

We excluded letters for drug-device combination NDAs and other application types, such as abbreviated new drug applications, biologic licensing applications, and non-efficacy supplements. A single refuse-to-file (RTF) letter that addressed multiple submissions was considered as separate letters for this analysis. Further, one application or supplement may receive multiple; each RTF is a separate entity and is analyzed accordingly in this study. A previously published methodology evaluating FDA "complete response letters" – letters that catalog reasons for not approving an application – was adapted for this study.[1]

Collection of RTFs

An internal FDA database (DARRTS) was used to collect RTFs. This database contains unredacted, complete review documents for NDAs and supplements to NDAs. For certain applications that have been approved, FDA posts the approval package (sometimes referred to as the "review package" or "action package"), which includes any RTFs that the FDA sent to the company, to its website at Drugs@FDA (https://www.accessdata.fda.gov/scripts/cder/daf/). However, the documents posted on this site are redacted according to applicable laws and regulations governing disclosure of confidential commercial or trade secret information. The documents we used for this study were not redacted.

© 2021 Chahal HS et al. *JAMA Internal Medicine*.

Collection of RTF characteristics

We collected the following variables from Document Archiving, Reporting & Regulatory Tracking System (DARRTS) for each RTF and its content:

Application/Supplement Number
Product Name: Brand
Product Name: Generic
Applicant
Applicant Size: Large/Small
Applicant Public/Private
Application Receive Date
RTF Issuance Date
Resubmission Date, if any
Most Recent Regulatory Status information as of July 2019
- Regulatory Status (e.g., review pending, approved or tentatively approved, complete response letter, withdrawn)
- Regulatory Status Date

Non-refuse-to-file Deficiencies Cited in Letter – Yes/No

Domains – Primary Reason(s) for refuse-to-file or non-refuse-to-file Content
- General Scientific and Technical
- Application Organization and Legal
- Chemistry, Manufacturing, and Controls
- Clinical Efficacy
- Clinical Safety
- Clinical Pharmacology and Biopharmaceutics
- Non-Clinical
- “Other” – non-refuse-to-file reasons

Sub-Domains – Specific Reasons for refuse-to-file (or Non- refuse-to-file Content) – See **eTable 1**

Application filed-over-protest – Yes/No
- Date filed-over-protest

Applicants were classified as “small” or “large” based on the U.S. Small Business Administration cutoff of 1,250 employees for pharmaceutical preparation manufacturing.[2] We used applicants’ websites, applicant-issued investor materials, Bloomberg Symbol Lookup, and SEC filings to determine whether applicants were publicly traded or headquartered in the U.S.[3, 4] We searched publicly available sources such as LinkedIn and US Securities and Exchange Commission (SEC) filings to estimate employee numbers. In this study, the application “owner” was defined as the applicant submitting the application or issuing the PR. If the submitting applicant was a subsidiary, the parent company was considered the “owner.”

Categorizing RTF reasons and other FDA comments

eTable 1 below shows the domains and subdomains used to categorize refusal reasons.
To assign a refusal reason to a domain and subdomain, we adhered closely to the reviewer’s terminology and letter organization. For example, if the reviewer did not use the term “safety” or “efficacy,” or the reason did not fall under a “safety” or “efficacy” heading, we did not assign the reason to the “Clinical Safety” or “Clinical Efficacy” domains. Thus, a reviewer statement that the risk/benefit analysis was inadequate, or the patient population was inappropriate, was assigned to the “General Scientific and Technical” domain, even though such statements may imply concerns with the reviewability of safety and/or efficacy.

© 2021 Chahal HS et al. *JAMA Internal Medicine*.

FDA's non-refuse-to-file comments did not necessarily critique applications on scientific merits, such as trials. Further, while comments on ignoring pre-submission requests/advice were not a reason for refusal, the ignored requests/advice may have resulted in an incomplete application. **eTable 2** shows other FDA comments in the RTFs that were not refusal reasons. **eTable 3** lists the specific types of FDA requests/advice that the applicant did not follow in the submission.

Determining applicants' public disclosure of RTFs

To identify PRs, we searched drug names, with and without "refuse" or "refusal" terms on applicants' websites, websites known to disseminate PRs, and Google searches.[5-8] The search timeframe extended from RTF issuance dates to July 2019. For publicly-traded applicants, SEC's Electronic Data Gathering, Analysis, and Retrieval system was searched to determine whether RTFs and their contents had been disclosed in securities filings.[4]

For public domestic firms (i.e., headquartered in the U.S.), we searched EDGAR chronologically for Forms '8-K,' '10-Q,' and '10-K.'[9-11] For foreign firms (non-U.S. based headquarters) traded on U.S. stock exchanges, we searched for Forms '6-K' and '20-F,' which correspond to Forms 8-K and 10-K for domestic firms, respectively.[12, 13]

Determining FDA's public disclosure of RTFs in approval packages

FDA discloses approval packages in accordance with applicable requirements and agency policies. Those requirements and policies govern what information is included in approval packages, which types of approval packages are posted, and when approval packages are posted. FDA posts the approval packages to the public Drugs@FDA database. We searched for each approved application and efficacy supplement to determine whether FDA had posted an approval package according to its applicable requirements and policies. If an approval package had been posted, we searched the documents for RTF letters, which are typically available in the "Administrative Document(s) & Correspondence" section.. **eTable 4** below shows the RTFs we included in this study that FDA made available on its website (Drugs@FDA) as of January 2020. These letters are redacted for non-public information. The letters used for analysis in this study were unredacted, available only to FDA investigators.

Post-hoc adjustment to domains

In a post-hoc adjustment, we split one domain into two for clarity. The data presented in the General Scientific and Technical, and the Application Organization and Legal domains were original collected under an umbrella domain called "General." The underlying data used to code the RTF reasons into the sub-domains were not changed.

Quality check and data analysis

Two investigators independently coded all RTFs, PRs, and SEC filings. The investigators had a 91% convergence in coding; discrepancies were resolved by consensus. Days to disclosure were measured in median days with interquartile range (IQR). Data were analyzed in Excel 365 (Microsoft Corp., Redmond, WA) and Python 3. Relative risk calculations, with 95% confidence intervals (95% CI), were conducted using SAS v9.4 (SAS Institute Inc., Cary, NC).

© 2021 Chahal HS et al. *JAMA Internal Medicine*.

eTable 1. Domains and Sub-Domains Used to Classify the Contents of FDA-Issued RTFs

Domain	*Sub-Domain – Full text used for coding FDA refusal-to-file reasons*	*Sub-domain – Short text used for data presentation*
Application Organization and Legal	1: Application not organized or well organized, legible, in English, or filed using Common Technical Document or according to FDA regulations/guidance(s)	*Application organization/reviewability*
	2: Inappropriate regulatory submission pathway (e.g., filed as 505(b)(1) instead of 505(j) or 505(b)(2), etc.) or inappropriate Reference Listed Drug	*Inappropriate regulatory submission pathway*
	3: Missing or inadequate patent information or certification; or exclusivity issues prevent review	*Patent certification or exclusivity issues*
	4: Missing or inadequate financial disclosure information	*Financial disclosure deficiencies*
General Scientific and Technical	1: Inappropriate patient population, design, or missing elements of any submitted study	*Inappropriate study design or patient population (any study)*
	2: Labeling materials/data/information missing or inadequate	*Deficient/requires labelling materials/data*
	3: Missing or inadequate pediatric information, including pediatric population study or plan	*Pediatric study/plan deficiencies*
	4: Study listings, data sets, metadata, coding, statistical analysis plan, or variable definitions not provided, incomplete, in non-standard format, or otherwise deficient	*Data sets and analytical plan deficiencies*
	5: Missing or inadequate clinical study reports (CSRs)	*Clinical study reports (CSRs) deficiencies*
	6: Missing or inadequate rationale for assuming applicability of foreign data	*Applicability of foreign data*
	7: Concerns about reliability of clinical study data; or missing, inadequate, or requires information on trial conduct	*Trial conduct and data integrity concerns*
	8: Missing, inadequate, or requires environmental analysis	*Environmental analysis deficient*
	9: Missing, inadequate, or requires benefit/risk analysis of the drug	*Benefit/risk analysis deficiencies*
	10: Pre-IND deficiencies identified	*Pre-IND deficiencies*
Clinical Efficacy	1: Missing adequate and well controlled clinical study for efficacy/requires new efficacy trial	*Missing/requires clinical study for efficacy*
	2: Missing, inadequate, or requires new reports, analyses, summaries, or data related to efficacy	*Missing/requires data analyses, reports, summaries for efficacy*
	3: Missing, inadequate, or requires new dose/frequency data for efficacy	*Missing/requires dose/frequency data for efficacy*
	4: Sample size for submitted efficacy study insufficient	*Insufficient efficacy study sample size*
	5: Chosen endpoints not clinically relevant, adequate, or appropriate	*Inappropriate efficacy clinical endpoints*

© 2021 Chahal HS et al. *JAMA Internal Medicine*.

	6: Analyzed outcome is not primary or protocol-specified (ad-hoc) or otherwise inappropriate	*Inappropriate efficacy outcome analysis*
	7: Missing, inadequate, or requires new supporting literature review or meta-analysis for efficacy	*Deficient/requires literature review/meta-analysis for efficacy*
Clinical Safety	1: Missing adequate and well controlled clinical study for safety/requires new safety study	*Missing/requires clinical study for safety*
	2: Missing, inadequate, or requires new reports, analyses, summaries, post-market analyses, or data related to safety	*Missing/requires data analyses, reports, summaries for safety*
	3: Missing, inadequate, or requires new dose/frequency data for safety	*Missing/requires dose/frequency data for safety*
	4: Missing, inadequate, or requires new supporting literature review or meta-analysis for safety	*Deficient/requires literature review/meta-analysis for safety*
Chemistry, Manufacturing and Controls	1: Missing, inadequate, or requires new methods, assay, data, study, or validation information on CMC processes	*Deficient/requires new CMC processes*
	2: Missing, deficient, or requires new data, studies, or other information on the investigated or commercial pharmaceutical product	*Deficient/requires new drug product information*
	3: Drug facility status unknown or not inspected, available, or ready for FDA inspection	*Facility status unknown or not ready for inspection*
	4: Drug Master Files not identified, not acceptable, incomplete, or not submitted to/received by FDA	*Deficient drug master files*
Clinical Pharmacology and Biopharmaceutics	1: Missing, inadequate, or requires new CP/PK/PD/BP data, information, summary, data, analyses, or study	*Deficient/requires CP/PK/PD/BP data/study*
	2: CP/PK/PD/BP study methods missing, incomplete, or otherwise deficient	*Deficient/requires CP/PK/PD/BP study methods*
	3: Missing, inadequate, or requires new drug interaction studies or data	*Deficient/requires drug interaction data/study*
	4: Missing, inadequate, or requires new dose adjustment studies or data	*Deficient/requires dose adjustment data/study*
	5: Missing, inadequate, or requires new abuse potential information, study, or data	*Deficient/requires abuse potential data/study*
Non-Clinical	1: Missing, inadequate, or requires new non-clinical pharmacology/PK summary, report, study, or data	*Deficient/requires non-clinical data/study*
	2: Missing or unsatisfactory pharmacology/toxicology studies or data	*Deficient/requires pharmacology/toxicology data/study*
	3: Missing or unsatisfactory carcinogenicity studies or data	*Deficient/requires carcinogenicity data/study*

© 2021 Chahal HS et al. *JAMA Internal Medicine*.

	4: Missing or unsatisfactory reproductive/teratogenicity studies or data	*Deficient/requires reproductive/teratogenicity data/study*
	5: Missing or unsatisfactory genotoxicity studies or data	*Deficient/requires genotoxicity data/study*

© 2021 Chahal HS et al. *JAMA Internal Medicine*.

eTable 2. Categories of Non-Refuse-to-File Comments FDA Made in RTFs

	Sub-Domain – Full text used for coding FDA's non-refusal-to-file comments	***Sub-domain – Short text used for data presentation***
Non-refuse-to-file comments by FDA	1: RTF discussed FDA recommendations/advice/requests in pre-submission meetings, previous RTFs, or other communications that the applicant did not follow or provide when submitting	*FDA requests/advice not followed**
	2: FDA provided a list of application deficiencies not related to refuse-to-file reasons	*FDA identified non-refuse-to-file deficiencies for resolution during full review*
	3: FDA recommended consulting experts in application prior to resubmission	*FDA recommended specialized consultation to improve application*
	4: FDA recommended a meeting with its reviewers to address deficiencies	*FDA recommended meeting with its reviewers*
	5: FDA referenced other trials or studies in similar therapeutic area for comparison	*FDA referenced/compared drug to others in similar therapeutic area*
*See eTable 3 for a detailed list of sub-categories		

© 2021 Chahal HS et al. *JAMA Internal Medicine*.

eTable 3. Types of FDA Presubmission Requests or Advice Not Followed by Applicants

Non-refuse-to-file comment category	Type of pre-submission FDA advice
FDA requests/advice not followed	Abuse potential studies needed
	Appropriate coding of datasets needed
	Clinical endpoint in trial(s) inappropriate
	Facility readiness for inspection
	FDA-recommended meeting with FDA reviewers
	Inappropriate patient population in clinical trial(s)
	Missing safety information or data needed
	Pre-IND deficiencies need to be addressed
	Supporting literature review needed
	Appropriate application organization needed
	Clinical pharmacology studies inadequate or need new studies
	Safety data or analysis inadequate or needed
	Non-clinical studies inadequate or need new studies
	Drug product chemistry and/or manufacturing information needed
	Clinical trial design inappropriate or inadequate or need new trial(s)

© 2021 Chahal HS et al. *JAMA Internal Medicine*.

eTable 4. Redacted RTFs Included in This Study That FDA Made Publicly Available as of January 2020

RTF #	Application #	Established Drug Name	Review Page #s	Link to FDA-posted approval packages containing RTFs
1	208078	Amifampridine Phosphate	19-23	https://www.accessdata.fda.gov/drugsatfda_docs/nda/2018/208078Orig1s000Admincorres.pdf
2	208901	Itraconazole	20-25	https://www.accessdata.fda.gov/drugsatfda_docs/nda/2018/208901Orig1s000AdminCorres.pdf
3	209964	Ivabradine	2-6	https://www.accessdata.fda.gov/drugsatfda_docs/nda/2019/209964Orig1s000Admincorres.pdf
4	22250	Dalfampridine	62-64	https://www.accessdata.fda.gov/drugsatfda_docs/nda/2010/022250s000_AdminCorres.pdf
5	22305	Purified Water	76-81	https://www.accessdata.fda.gov/drugsatfda_docs/nda/2011/022305Orig1s000Admincorres.pdf
6	22434	Argatroban	112-114	https://www.accessdata.fda.gov/drugsatfda_docs/nda/2011/022434Orig1s000AdminCorres.pdf
7	22512	Dabigatran Etexilate Mesylate	45-47	https://www.accessdata.fda.gov/drugsatfda_docs/nda/2010/022512Orig1s000Admin.pdf
8	22561	Cladribine	20-28	https://www.accessdata.fda.gov/drugsatfda_docs/nda/2019/022561Orig1s000AdminCorres.pdf
9	200153	Atorvastatin and Ezetimibe	70-72	https://www.accessdata.fda.gov/drugsatfda_docs/nda/2013/200153Orig1s000AdminCorres.pdf
10	201635	Topiramate	151-157	https://www.accessdata.fda.gov/drugsatfda_docs/nda/2013/201635Orig1s000AdminCorres.pdf
11	201849	Glucagon	125-128	https://www.accessdata.fda.gov/drugsatfda_docs/nda/2015/201849Orig1s000AdminCorresd.pdf
12	201922	Minocycline Hydrochloride	73-75	https://www.accessdata.fda.gov/drugsatfda_docs/nda/2012/201922Orig1s000Admincorres.pdf
13	202834	Perampanel	217-230	https://www.accessdata.fda.gov/drugsatfda_docs/nda/2012/202834Orig1s000AdminCorres.pdf
14	203231	Zoledronic Acid	103-105	https://www.accessdata.fda.gov/drugsatfda_docs/nda/2013/203231Orig1s000Admincorres.pdf
15	203324	Riboflavin 5'-Phosphate, Photrexa® Riboflavin 5'-Phosphate, And Kxl™ System	175-180	https://www.accessdata.fda.gov/drugsatfda_docs/nda/2016/203324Orig1s000Admincorres.pdf
16	203510	Phenylephrine Hydrochloride	35-37	https://www.accessdata.fda.gov/drugsatfda_docs/nda/2013/203510Orig1s000Admincorres.pdf
17	203856	Cyclophosphamide	134-136	https://www.accessdata.fda.gov/drugsatfda_docs/nda/2013/203856Orig1s000Admincorres.pdf
18	203952	Carbidopa and Levodopa	83-88	https://www.accessdata.fda.gov/drugsatfda_docs/nda/2015/203952Orig1s000AdminCorres.pdf
19	204684	Miltefosine	177-180	https://www.accessdata.fda.gov/drugsatfda_docs/nda/2014/204684Orig1s000AdminCorres.pdf
20	205525	Dronabinol	75-78	https://www.accessdata.fda.gov/drugsatfda_docs/nda/2016/205525Orig1s000AdminCorres.pdf
21	206976	Diclofenac Epolamine, and Heparin	2-7	https://www.accessdata.fda.gov/drugsatfda_docs/nda/2018/206976Orig1s000AdminCorres.pdf
22	207145	Safinamide Mesylate	65-70	https://www.accessdata.fda.gov/drugsatfda_docs/nda/2017/207145Orig1s000AdminCorres.pdf

© 2021 Chahal HS et al. *JAMA Internal Medicine*.

23	207964	Chlorhexidine Gluconate	2-7	https://www.accessdata.fda.gov/drugsatfda_docs/nda/2018/207964Orig1s000AdminCorres.pdf
24	208025	Lansoprazole	51-55	https://www.accessdata.fda.gov/drugsatfda_docs/nda/2016/208025Orig1s000Admincorres.pdf
25	208042	Buprenorphine and Naloxone	2-5	https://www.accessdata.fda.gov/drugsatfda_docs/nda/2018/208042Orig1s000Admincorres.pdf
26	208144	Brimonidine Tartrate	53-56	https://www.accessdata.fda.gov/drugsatfda_docs/nda/2017/208144Orig1s000AdminCorres.pdf
27	209184	Levodopa	2-6	https://www.accessdata.fda.gov/drugsatfda_docs/nda/2018/209184Orig1s000AdminCorres.pdf
28	210607	Tafenoquine	16-20	https://www.accessdata.fda.gov/drugsatfda_docs/nda/2018/210607Orig1s000AdminCorres.pdf
29	22404	Miconazole	149, 151	https://www.accessdata.fda.gov/drugsatfda_docs/nda/2010/022404Orig1s000AdminCorres.pdf

© 2021 Chahal HS et al. *JAMA Internal Medicine*.

eReferences

1. Lurie, P., et al., *Comparison of content of FDA letters not approving applications for new drugs and associated public announcements from sponsors: cross sectional study.* The British Medical Journal (BMJ), 2015. **350**(h2758) DOI: 10.1136/bmj.h2758.
2. U.S. Small Business Administration. *Table of Small Business Size Standards*. 2017 [cited 2017 July 24]; Available from: https://www.sba.gov/contracting/getting-started-contractor/make-sure-you-meet-sba-size-standards/table-small-business-size-standards.
3. Bloomberg LP. *Stocks*. 2017 [cited 2017 July 26]; Available from: https://www.bloomberg.com/markets/stocks.
4. U.S. Securities and Exchange Commission (SEC). *Electronic Data Gathering, Analysis, and Retrieval System (EDGAR). Company search*. 2017; Available from: www.sec.gov/edgar/searchedgar/companysearch.html.
5. PR Newswire Association LLC. *PR Newswire: press release distribution, targeting and monitoring services*. 2017 [cited 2017 August 27]; Available from: http://www.prnewswire.com/.
6. Questex LLC. *FiercePharma*. 2017 [cited 2017 August 27]; Available from: http://www.fiercepharma.com/.
7. Questex LLC. *FierceBiotech*. 2017 [cited 2017 August 27]; Available from: http://www.fiercebiotech.com/.
8. Business Wire Inc. *Business Wire*. 2017 [cited 2017 August 27]; Available from: http://www.businesswire.com/portal/site/home/.
9. *Form 10-Q, for quarterly and transition reports under sections 13 or 15(d) of the Securities Exchange Act of 1934. 17 CFR 249.308a*.
10. *Form 10-K, for annual and transition reports pursuant to sections 13 or 15(d) of the Securities Exchange Act of 1934. 17 CFR 249.310*.
11. *Form 8-K, for current reports. 17 CFR 249.308*.
12. *Form 6-K, report of foreign issuer pursuant to Rules 13a-16 (§ 240.13a-16 of this chapter) and 15d-16 (§ 240.15d-16 of this chapter) under the Securities Exchange Act of 1934. 17 CFR 249.306*.
13. US Securities and Exchange Commission. *Fast Answers - Form 8-K, Form 10-K, Form 10-Q*. 2017 [cited 2017 August 27]; Available from: https://www.sec.gov/fast-answers/.

© 2021 Chahal HS et al. *JAMA Internal Medicine*.
